# The Role of Connexin Channels in the Response of Mechanical Loading and Unloading of Bone

**DOI:** 10.3390/ijms21031146

**Published:** 2020-02-09

**Authors:** Manuel A. Riquelme, Eduardo R. Cardenas, Huiyun Xu, Jean X. Jiang

**Affiliations:** 1 Department of Biochemistry and Structural Biology, University of Texas Health Science Center, San Antonio, TX 78229, USA; Riquelme@uthscsa.edu (M.A.R.); CardenasE2@uthscsa.edu (E.R.C.); 2 Key Laboratory for Space Bioscience and Biotechnology, School of Life Sciences, Northwestern Polytechnical University, Xi’an 710072, China; celldon@nwpu.edu.cn

**Keywords:** bone remodeling, mechanical loading, osteocytes, mechano-transduction, connexin43, hemichannels

## Abstract

The skeleton adapts to mechanical loading to promote bone formation and remodeling. While most bone cells are involved in mechanosensing, it is well accepted that osteocytes are the principal mechanosensory cells. The osteocyte cell body and processes are surrounded by a fluid-filled space, forming an extensive lacuno-canalicular network. The flow of interstitial fluid is a major stress-related factor that transmits mechanical stimulation to bone cells. The long dendritic processes of osteocytes form a gap junction channel network connecting not only neighboring osteocytes, but also cells on the bone surface, such as osteoblasts and osteoclasts. Mechanosensitive osteocytes also form hemichannels that mediate the communication between the cytoplasmic and extracellular microenvironment. This paper will discuss recent research progress regarding connexin (Cx)-forming gap junctions and hemichannels in osteocytes, osteoblasts, and other bone cells, including those richly expressing Cx43. We will then cover the recent progress regarding the regulation of these channels by mechanical loading and the role of integrins and signals in mediating Cx43 channels, and bone cell function and viability. Finally, we will summarize the recent studies regarding bone responses to mechanical unloading in Cx43 transgenic mouse models. The osteocyte has been perceived as the center of bone remodeling, and connexin channels enriched in osteocytes are a likely major player in meditating the function of bone. Based on numerous studies, connexin channels may present as a potential new therapeutic target in the treatment of bone loss and osteoporosis. This review will primarily focus on Cx43, with some discussion in other connexins expressed in bone cells.

## 1. Connexin 43 Gap Junctions, Hemichannels and Bone Homeostasis

Gap junctions serve as a conduit for signal transduction between bone cells, which allow metabolic and electric coupling for an efficient response to extracellular stimuli. Gap junctions are clusters of channels that allow for cytoplasmic connections between cells. These junctions are formed by the docking of two connexins (hemichannels) provided by each of the neighboring cells [1,2]. Each hemichannel is composed of six connexin (Cx) molecules that are named according to their predicted molecular weight. To date, 21 different human connexins have been identified.

Gap junctions are permeable to molecules below 1 kDa, which includes, ions, such as, K^+^, Na^+^, Ca^2+^, and metabolites, lactate, glucose 6 phosphate and signaling molecules, cAMP, IP_3_, and ATP. Hemichannels—unopposed halves of gap junctions—connect the cell cytoplasm with the extracellular microenvironment. They share a similar permeability with gap junction channels. The opening of hemichannels allows for the release of molecules that are in a high concentration in the cytoplasm, including, ATP, PGE_2_, NAD^+^, glutathione, glutamate, and K^+^ [3]. Intracellular domains of connexins, like the intracellular loop and C-terminal tail, are sites for interaction and stabilization of kinases, the cytoskeleton, adhesion molecules, regulatory proteins, and transcription factors [4]. The lack of specific tools to discriminate the role of gap junctions, hemichannels, and channel/hemichannel-independent functions limits the interpretation of the phenotypes associated with connexin genetic deletion animal studies. 

**Figure 1:**
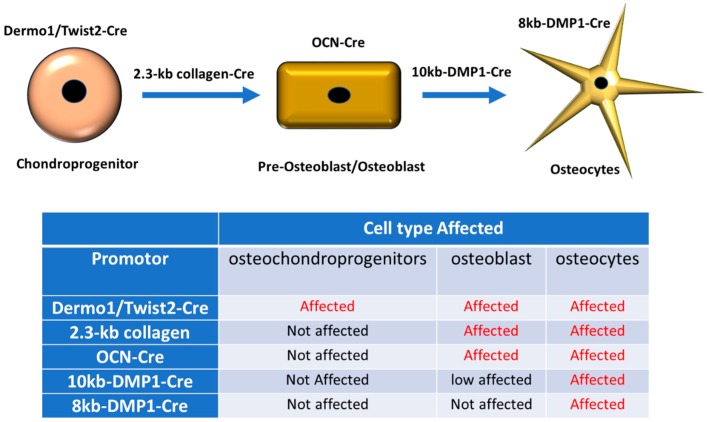
Schematic representation of sequential activation of bone master gene promoters used for Cre expression or gene overexpression. During bone development there is a successive expression of master genes that provide the special characteristics of bone cells. For example, chondroprogenitor cells express the Dermo1/Twist2 promotor, if fused with Cre protein and expressed in the cell, flox-tagged genes will be removed and the complete cell linage will be affected. However, if Cre is controlled by a late gene such as DMP1, which is mainly expressed in osteocytes, flox-tagged genes will be primarily removed in osteocytes and not preceding cell lineage types, such as the osteoblasts. The promoters mentioned in this review and the differentiation state for their activation are summarized in the table below.

Several connexin proteins have been found in bone cells, such as, Cx37, Cx43, and Cx45 in osteoblasts and osteocytes, and Cx37 and Cx43 in osteoclasts. Global deletion of Cx37 resulted in increased bone mass due to decreased osteoclast differentiation and bone resorption without altering osteoblast differentiation or function. This effect was more pronounced in male rather than in female mice [5]. Cx43 is the most abundantly expressed and studied connexin subtype in bone [6]. The first conventional connexin gene knockout study in mice has been reported for Cx43, but the mice die of asphyxiation due to heart malformation shortly after birth [7]. These mice exhibit delayed ossification, and osteoblasts isolated from these Cx43 knockout (KO) mice show compromised osteoblast differentiation [8]. Mice with deletion of Cx43 in the osteoblastic cell lineage are viable and exhibit bone phenotypes that are progressively severe. Cx43 deletion in bone precursor cells is more severe compared to cells of later osteoblastic lineage [9,10,11,12]. In fact, deletion of Cx43 from osteochondroprogenitors using the Dermo1/Twist2 promoter to target Cre recombinase expression (Figure 1) leads to phenotypes with decreased bone mass and a reduced length of long bones [9]. Cx43 deletion in committed osteoblast progenitors using a 2.3-kb collagen promoter fragment driving Cre (Figure 1) results in reduced bone mineral density, yet body size, compared with their WT littermates, remains the same [10]. Deletion of Cx43 in mature osteoblasts using the human osteocalcin promoter to drive Cre expression or in osteocytes using the 8-kb DMP-1 promoter (Figure 1) does not affect longitudinal bone growth, and bone mineral density [13,14]. Cx43 deletion in the osteoblastic cell lineage results in increased periosteal bone formation and endocortical bone resorption, with a wide bone marrow cavity and external perimeter of long bones [9,12,15]. These mice display increased cortical osteocyte apoptosis [14], periosteal bone formation, and a high RANKL/OPG ratio, which favors osteoclast differentiation and bone resorption [9,12,14]. Interestingly, C-terminal truncation of Cx43 (Gja1 K258Stop) exhibits remarkable cortical bone phenotypes similar to the deletion of the entire Cx43 gene in osteoblasts [16]. In this paper, they also report the interaction of Cx43 C-terminus with several signaling proteins, including the known pro-bone forming Wnt signaling molecule, β-catenin. Conversely, a recent study shows that Cx43 overexpression in osteocytes (8kb-DMP1-Cre-Cx43/GPF) ameliorates age related cortical bone changes, such as increased osteocyte viability, that helps maintain formation, and improves bone strength compared to wild type mice [17].

The specific role of gap junction channels and hemichannels formed by Cx43 was identified using two transgenic mouse models driven by a 10 kb DMP-1 promoter (Figure 1) and expressing two dominant-negative Cx43 mutants [18]. Cx43 Δ130-136 with a short truncation mutation in the cytoplasmic loop region that dominant negatively inhibited gap junctions and hemichannels, and R76W with a site mutation on the second transmembrane domain only plays a dominant negative role in gap junctions. Significant changes were observed in bone mass, structure, strength, and osteocyte viability in Cx43 Δ130-136 mice. While the overall bone phenotype in Cx43R76W mice is less evident, relative to wild type, the mice show increased endocortical osteoclast numbers, activity, and the expression of serum bone remodeling markers. The data from these two transgenic models suggest that Cx43 hemichannels in osteocytes likely play a predominant role in osteocyte function. This was evident from the major impact on the expression of OPG and RANKL, which are two key proteins involved in osteoclastogenesis and osteocyte viability and is essential for bone integrity and longevity. However, gap junctions are more involved in the global regulation and fine tuning of bone formation and resorption, which are implicated for the alterations of serum remodeling markers N-terminal propeptide of type I procollagen (PINP) and C-terminal telopeptide of type I collagen (CTX) [18].

Mutations in the Cx43 gene are associated with *oculodentodigital dysplasia* (ODDD) [19] and *craniometaphyseal dysplasia* [20] in humans. These abnormalities have been reproduced in two mouse gene knock-in models in which the Cx43 gene was replaced with Cx43G138R or Cx43G60S mutants [21,22,23]. Expression of Cx43G138R under Dermo1/Twist2 promotor in the chondro-osteogenic lineage recapitulates the skeletal phenotype of mice with a global expression of the mutated gene [9]. Studies show that, besides decreased bone mass, mice expressing Cx43G60S, a dominant-negative mutant that disrupts the gap junction assembly and function, exhibits changes in the bone marrow with progressive bone marrow atrophy and increased adipocytes [23,24]. These phenotypic changes were not reported for mice carrying the Cx43G138R ODDD mutation, which does not alter gap junction assembly, but impairs the gap junction function with leaky hemichannels [21,25].

## 2. Mechanical Loading Signaling in the Bone and Involvement of Connexins and Pannexins

Physical activity, such as exercise, results in bone mechanical loading, which induces the movement of interstitial fluid within bone. Shear stress is sensed by the osteocytes through several components, including integrins, cilia, calcium channels, and G-protein coupled receptors. The above factors function as mechano-sensors of bone. It has been suggested that strains in bone are not constant [26], with physiological loads estimated to be in the range of 8-30 dyn/cm^2^, and these factors allow for a finely tuned response to mechanical loading [27]. Tethering elements, like integrin proteins, which attach and anchor the dendrites to the canalicular walls and mineralized matrix, allow the dendritic tips to interact with other osteocyte dendrites and form gap junction channels. These intercellular channels permit fast cell-to-cell communication in order to respond to extracellular stimuli [28]. Due to the unique tethering elements, osteocyte processes are extremely responsive to pico newton-level mechanical loading in the cell dendrites. Integrins αvβ3 and α5β1 integrins are highly expressed in osteocytes and connect the intracellular actin cytoskeleton to extracellular matrix components, such as the glycocalyx, fibronectin, vitronectin, and osteopontin [29,30]. The osteocyte cell body and processes lacking local attachments to the extracellular matrix are less responsive to mechanical loading [31]. Dissociation of the cell matrix or inhibition of αvβ3 integrin attachment sites disrupts the response to mechanical stimulation [29,31]. Integrin α and β heterodimers is also shown to respond to fluid flow shear stress, which induces conformational changes in the β-subunit, resulting in the activation of a bone signaling cascade [30].

The actin cytoskeleton transmits mechanical forces from one focal adhesion site to another mechano-sensing site within the cell and to neighboring cells. Focal adhesions are complexes of several proteins that allow for communication between the cell and the extracellular environment, serving as a mechanical linkage between the ECM and cytoskeleton, and as a site for signal transduction. One example of how focal adhesions function to transmit signals is focal adhesion kinase (FAK). FAK localizes to focal adhesions, and is required for osteocyte mechanotransduction by coordinating with integrins that lead to the activation of downstream targets including changes in gene expression through the src kinase [32].

Spectrin, a structural cytoskeletal protein, is required for the differentiation of osteoblasts to osteocytes, and has been identified as a mechanosensitive element within the osteocyte [33]. Disruption of the spectrin network increases clustered Cx43 gap junction plaques, and promotes persistent Ca^2+^ influx and enhanced nitric oxide (NO) secretion, resulting in reduced cell stiffness [33].

Contrary to the predicted anabolic role of Cx43, Cx43 KO in osteochondroprogenitors (Cx43^fl/fl^; Dermo1-Cre) results in an exaggerated anabolic response on the periosteal surface of the tibia [34]. Furthermore, an enhanced anabolic response to mechanical loading has been observed on the periosteal surface in mice lacking Cx43 in mature osteoblasts and osteocytes (Cx43^fl/fl^;OCN-Cre) [12]. Similarly, deletion of Cx43 in osteocytes (Cx43^fl/fl^; DMP1-Cre) is sufficient to enhance the anabolic response to mechanical loading on the periosteal surface [11]. These results suggest that Cx43 during mechanical loading may function as an inhibitor of the anabolic response at the periosteal surface. However, deletion of Cx43 in Cx43^fl/fl^ Dermo1-Cre mice results in decreased bone formation, including mineralization surface and bone forming rate on the endocortical surface [34]. However, mechanical stimulation does not decrease bone formation on the endocortical surface in the presence or absence of Cx43 in osteocytes [11], suggesting that Cx43 expression in osteoblasts, but not osteocytes, is required to maintain endocortical bone formation under mechanical stimulation.

The Wnt signaling pathway is shown to function in bone homeostasis. Osteocytes maintain bone tissue by producing important bone regulators, such as Wnt ligands Wnt1 and mechanically stimulated Wnt3a [35,36]. Wnt molecules promote bone formation and increase the osteoblast number through the activation of the Wnt signaling pathway [37]. However, to maintain bone homeostasis, osteocytes also express the Wnt/β-catenin signal inhibitor sclerostin [38]. Loss-of-function mutations in the SOST gene (encoding sclerostin) cause osteosclerosis and *van Buchem* disease with abnormally high bone mass [39,40], suggesting that bone formation is usually suppressed by sclerostin through the inhibition of Wnt/β-catenin signals. Osteocytes and bone from mice lacking Cx43 in osteocytes exhibit increased expression levels of β-catenin and Wnt target genes, suggesting that, in the absence of Cx43, osteocytes are primed to respond to mechanical forces through the anabolic effects of the Wnt signaling pathway [11]. Indeed, studies by Moorer et al. directly link Cx43 with the Wnt signaling pathway [41]. More specifically, they identified β-catenin as binding to the C-terminal domain of Cx43, thus inhibiting Wnt-activated nuclear translocation of β-catenin and target gene activation [42]. β-catenin nuclear translocation can suppress the RANKL expression [43], however, there is no solid evidence demonstrating the direct interaction between Cx43 and β-catenin in bone cells. Together, Cx43, RANKL, Wnt, and β-catenin appear to be critical to the mechanism, by which bone cells respond to mechanical signals [44]. Moreover, deletion of one allele of β-catenin in β-catenin^+/−^ mice (Dmp1-Cre β-catenin fl/+; HET cKO) abolishes the anabolic response to mechanical loading, suggesting that a certain level of β-catenin is needed to provoke a response [45]. In addition, the levels of the Wnt inhibitor Sost/sclerostin and the proportion of osteocytes expressing Sost/sclerostin are decreased in several models of Cx43 deficiency [9,15,46], raising the possibility that the absence of the Wnt inhibitor might contribute to enhanced bone formation in both control and loaded bone. It is likely that the local bone formation in areas with a low number of osteocytes, likely due to the accumulation of apoptotic osteocytes, leads to low levels of sclerostin, and consequently increased bone formation [45,46]. However, alterations of cortical bone parameters, such as mineral density, thickness, width, and geometry, due to Cx43 deficiency, are not solely mediated by decreased sclerostin levels, as shown in the lack of cortical abnormalities in haplodeficient models of Sost and Cx43 [47]. While this intriguing result does not support the interaction of Cx43 and Sost in cortical bone modeling, it cannot exclude the possibility of altered regulation undetermined components in this double heterozygous deletion model. The haploid presence of Cx43 restores the channel function and compensates the bone phenotype to wild type. These results support the notion that absence of Wnt inhibition associated with increased bone formation could be a consequence of the reduced viability of osteocytes in mice without Cx43. The presence of the Cx43 hemichannel function rescues the bone phenotype, which further supports the importance of these channels [18,47].

While Cx43 is a predominant connexin expressed in bone cells, other connexin subtypes, as aforementioned, are also detected in bone cells. However, their roles in mechanosensing and mechanotransduction have not been reported. Pannexin1 (Panx1) is another player recently discovered to function in bone homeostasis. The bones of Panx1KO mice after loading express an upregulation of Sost and downregulation of β-catenin RNA messenger, suggesting the role of Panx1 in bone anabolic responses [48]. However, under skeletal fatigue loading, which induces microdamage and bone remodeling, the functional axis of Panx1/P2X7R is required to trigger the increased levels of RANKL in osteocytes without increasing apoptosis [48]. This model has recently been replicated in vitro.

## 3. The Role of Cx43 and Cx43 Channels in Mechanotransduction

While there have been many studies on Cx43, the mechanism by which Cx43 deletion enhances the anabolic response to loading is not well understood. As stated above, there is strong in vivo evidence that the Wnt signaling pathway is crucial in bone homeostasis, in which osteocytes are shown to produce key pro-bone formation Wnt molecules (Wnt1 and Wnt3a), as well as Wnt/β-catenin signaling inhibitors (sclerostin) [35,36,37,38]. Furthermore, heterozygous deletion of Dkk-1 (a Wnt inhibitor) in osteoblasts results in a decrease in bone mass [49,50]. Wnt signaling also affects bone homeostasis through the upregulation of OPG and down regulation of RANKL, the latter is a molecule required for osteoclast differentiation [51]. Data directly connecting the Wnt pathway and Cx43 include studies on the mouse osteocyte cell line MLO-Y4 under fluid flow shear stress, where mechanical loading increased the levels of Wnt3a, Cx43, OPG, and reduced the levels of RANKL and Dkk1, as previously mentioned [52,53].

**Figure 2:**
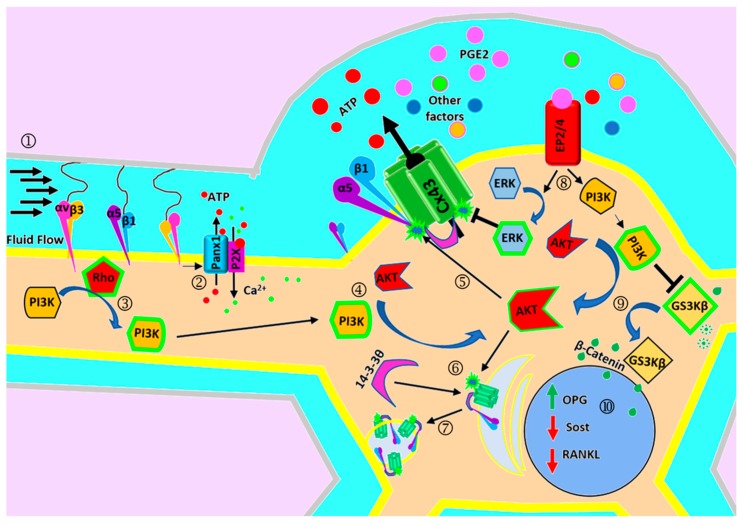
Connexin hemichannels in mechanotransduction of osteocytes. During FFSS, mechanical forces sensed by osteocytic dendrites are transduced through integrins, αvβ3 and α5β1 (①). αvβ3 integrins induce the fast activation of Panx1/P2XR (purinergic receptor) channels and an increase of intracellular Ca^2+^ concentration, which propagates as a Ca^2+^ wave (②). Moreover, αvβ3 integrin induces the activation of focal adhesion kinases, known activators of signal transduction pathways (③). Mechanical loading activates the AKT pathway (④), which promotes the opening of Cx43 hemichannels (⑤) and stabilizes Cx43/α5β1 integrin/14-3-3θ complex, and 14-3-3θ (⑥) facilities the translocation of Cx43 and α5β1 integrin to the cell surface (⑦). The opening of hemichannels leads to a release of factors that promote bone formation and osteocyte survival signaling. For example, PGE_2_ released by Cx43 hemichannels activates EP2/4 (prostaglandin receptor) that induces the activation of PI3K singling pathway (⑧), which leads to inhibition of GSK3β (⑨), allowing the accumulation of β-catenin required to modulate bone remodeling genes, such as OPG, RANKL and Sost (⑩). Moreover, EP2/4 activates the ERK signaling pathway and through direct protein phosphorylation of Cx43, promotes the closure of hemichannels (⑧).

Osteocyte cell bodies reside in small cavities known as lacunae, and multiple long dendritic processes branching from the cell body travel in the bone mineral matrix inside canals termed canaliculi that form a network with other osteocytes and bone cells. The flow of fluid in the bone lacunar-canalicular network is a major source triggering a intracellular Ca^2+^ response to the mechanical stimulation applied to osteocytes [54]. In another study, fluid flow shear stress induced the opening of Cx43 hemichannels in mouse primary osteocytes and MLO-Y4 cells [55,56]. These observations suggest that the mechanism of sensing and opening of Cx43 hemichannels by mechanical loading in primary cells and established cell lines is likely conserved. Shear stress forces sensed in the dendrites require the interaction of dendritic elements such as integrins with the glycocalyx matrix to increase the activity of Cx43 hemichannels that are abundantly expressed in the cell body [29]. Shear stress activates αvβ3 integrins that are present in dendrites, and promotes a fast activation of Panx1 channels and ATP release, which activates purinergic pathways crucial for bone homeostasis (Figure 2) [31]. The activation pathway of Cx43 hemichannels in the osteocytic cell body remains largely unknown. Fluid flow shear stress enhances the interaction of integrin α5 with the C-terminal cytoplasmic domain of Cx43, which is required for the opening of Cx43 hemichannels [55]. Furthermore, antibody activation of β1 integrins induces the opening of Cx43 hemichannels in vitro, suggesting that the activation of integrins is sufficient for hemichannel opening. Moreover, Cx43 and integrin α5 interaction depends on the activation of AKT signaling pathways [57]. Cx43 phosphorylation in the AKT consensus site promotes the interaction of the scaffolding molecule, 14-3-3θ, which stabilizes the complex containing Cx43, 14-3-3θ and integrin α5 [58]. Reduction of the 14-3-3θ protein prevents the accumulation of Cx43 on the plasma membrane and the reduction of hemichannels activity triggered by fluid flow. The activation of α5 integrin using activating antibodies promotes the opening of Cx43 hemichannels, even under non-mechanical loading, i.e., under resting conditions (Figure 2) [55]. These results show that integrins are a major component of regulatory pathways that lead to the opening of Cx43 hemichannels under physiological conditions.

## 4. Regulation of Cx43 and Cx43 Channels by Mechanical Loading

The physiological role of Cx43 hemichannel activation induced by mechanical stress was characterized by molecules released by these channels. It is shown that shear stress induced by fluid flow leads to the release of PGE_2_ and ATP, both negatively charged molecules at physiological pH that are key in bone homeostasis. The release of both factors is sensitive to an unspecific connexin/pannexin channel chemical blocker, β-glycyrrhetinic acid, and specific inhibitors, Cx43 anti-sense oligonucleotides and Cx43 hemichannel blocker Cx43E2 antibody [56,59,60]. These results indicate that Cx43 hemichannels are involved in the release of signaling metabolites from osteocytes in response to mechanical stimulation. PGE_2_ release, following mechanical stimulation of MLO-Y4 cells, results in the activation of autocrine/paracrine signaling that contributes to cell survival [61]. Consistent with this study, fluid flow shear stress inhibits the glucocorticoid-induced apoptosis of MLO-Y4 cells, an effect that is reversed by inhibiting prostaglandin synthesis with indomethacin [62]. Furthermore, this study showed that the addition of PGE_2_ is sufficient to prevent osteocyte apoptosis by a mechanism that requires the activation of the cAMP/PKA and PI3K/Akt/β-catenin signaling pathways [62]. Continuous fluid flow shear stress induces the release and accumulation of PGE_2_ in a concentration range that can activate EP2/EP4 receptors and subsequently ERK activation, and the latter phosphorylates Cx43 in the MAPK consensus site at the carboxyl-terminal domain. The phosphorylation of Cx43 by MAPK promotes the closure and retrieval of Cx43 hemichannels on the cell surface (Figure 2) [56]. In addition, Cx43 hemichannel opening and concomitant activation of the ERK pathway induced by oxidative stress or bisphosphonates promote osteocyte survival [56,61,63,64]. These studies strongly suggest that mechanical stimulation activates integrins α5β1, which, in turn, activates FAK/Src and PI3K/AKT kinases. AKT phosphorylation of Cx43 stabilizes the open conformation of the channel, leading to the release of PGE_2_, and activation of EP2/EP4 receptors. Accumulated extracellular PGE_2_ activates the ERK pathway. ERK phosphorylation on Cx43, however, leads to the closure of Cx43 hemichannels. The specific phosphorylation of Cx43 by AKT and MAPK regulates opening and closing of hemichannels, respectively, which contributes to its role in the maintenance of osteocyte viability (Figure 2). The results obtained from these in vitro cell studies support the observation of increased apoptosis and empty lacunae in the cortical bone of osteocyte-specific Cx43 knockout and Cx43 transgenic mice.

We place special attention to Cx43 and the Wnt signaling pathway and the large body of evidence suggesting the role of Cx43 in bone signaling and its connection to the Wnt pathway. However, there are other key factors involved in mechanotransduction. For example, studies show that antisense oligonucleotides and the specific Cx43 hemichannel blocker Cx43E2 also inhibit PGE_2_ and ATP release. While Cx43 is our primary focus here, and there is much data supporting Cx43 in bone homeostasis and mechantransduction, it should not be surprising that other channels are at play. One study by Thi et al. suggests that Panx1-forming hemichannels along with P2X_7_R form a complex and are involved in mechanosensing in osteoblasts [65]. In this study, they found Cx43-null osteoblasts did not alter PGE_2_ release, contrary to osteocytes, in response to fluid shear stress. Therefore, we cannot overlook the significant roles of other mechano-sensors and mechanotransducers in bone and their functional interactions with connexin channels.

## 5. Role of Cx43 in Response to Mechanical Unloading/Disuse

Mechanical unloading/disuse is commonly experienced during spaceflight [66] and reduced physical activity [67], such as bed rest and spinal cord injury [68]. For bone tissue, the major consequence caused by unloading is bone loss, including the decrease of bone mass and BMD, which may be caused by decreased bone formation and increased bone resorption [69]. Studies show the functional involvement of Cx43-forming gap junctions and hemichannels in the process of mechanical unloading.

Bone loss caused by microgravity has been identified as one of the most serious physiological problems for astronauts. Matsuda et al. (2006) have shown that cell-to-cell communications via gap junctions in osteocytic MLO-Y4 cells are significantly decreased when cells are subjected to multi-dimensional gravity in a three-dimensional (3D) culture [70]. A previous study also showed that gravity changes decrease the expression of Cx43 in MLO-Y4 cells after 24 h of parabolic flight [71]. Furthermore, simulated microgravity by a random position machine (RPM) reduces the surface expression of Cx43, but increases the activity of Cx43 hemichannels and the release of PGE_2_ [72]. MLO-Y4 cells were also subjected to a high magneto-gravitational environment, and the role of Cx43 hemichannels in global gene expression was assessed using a specific blocking antibody Cx43(E2). In this study, a simulated weightlessness was obtained in which cells were placed at a position where the magnetic force could balance out the gravity, leading to stable diamagnetic levitation. Results show that blocking hemichannels significantly affects the expression of over 800 genes, including genes involved in cell viability, apoptosis, mineral absorption, protein absorption and digestion, and focal adhesions [72].

Several mouse models with Cx43 gene modification have been used to investigate the role of Cx43 in response to mechanical unloading. Grimston and colleagues treated male Col1α1-Cre; Cx43^-/flx^ mice with botulinum toxin A by injecting the drug into the hind limb muscle to generate a muscle paralysis unloading model. They found that osteoblast/osteocyte-specific Cx43 knockout showed less sensitivity to cortical bone loss, but a similar sensitivity in trabecular bone compared to wild-type mice [73]. Lloyd and colleagues found that Cx43 deficiency in male OCN-Cre;Cx43^flox/flox^ mice desensitized bone to the effects of mechanical unloading caused by hind limb suspension (HLS) in trabecular, but not in cortical bone [13]. This study implies the role of Cx43 in modulation of both arms of bone remodeling, bone formation, and bone resorption [15]. To unveil the specific roles of gap junctions and hemichannels, two transgenic mouse models are used. As discussed earlier, these two mouse models are driven by a 10 kb DMP1 promoter with the overexpression of dominant negative mutants of Cx43, Δ130-136, and R76W, which act as dominant negative inhibitors of gap junction and hemichannels, or gap junctions only, respectively [18]. These two mouse models were subjected to unloading using an HLS model to unveil the distinct roles of gap junctions and hemichannels. Our recent study showed that, compared to WT mice, both R76W and Δ130-136 transgenic mice had increased bone loss in the midshaft cortical area and a comparable level of trabecular bone loss. However, R76W and Δ130-136 mice showed differences in osteocyte viability and bone remodeling in response to HLS, implying that hemichannels and gap junctions play distinct roles in response to unloading (unpublished data).

## 6. Future Perspectives

Our mechanistic understanding and knowledge of osteocytes and gap junctions/hemichannels responding to mechanical loading have been exponentially increased in recent years, due to the development of new techniques, methodologies, and genetic animal models. However, the impact of mechano-activated osteocytes and connexin channels on other bone cells, the bone matrix microenvironment, bone marrow, and other cells/tissues adjacent to osteocytes remain largely unknown. These questions will be the direction of the next, exciting phase of bone research and will hold the most promise in future bone therapy. The detailed mechanistic studies will be integrated into broader skeletal and overall body physiology, which will help in the discovery and the development of new, safe pharmacological targets and strategies to treat bone diseases.
